# Improving the thermostability of GH49 dextranase AoDex by site-directed mutagenesis

**DOI:** 10.1186/s13568-023-01513-2

**Published:** 2023-01-19

**Authors:** Zhen Wei, Jinling Chen, Linxiang Xu, Nannan Liu, Jie Yang, Shujun Wang

**Affiliations:** 1 Jiangsu Key Laboratory of Marine Bioresources and Environment, Co-Innovation Center of Jiangsu Marine Bio-Industry Technology, Jiangsu Ocean University, Lianyungang, 222005 China; 2 School of Food Science and Engineering, Jiangsu Ocean University, Lianyungang, 222005 China; 3 Jiangsu Institute of Marine Resources Development, Jiangsu Ocean University, Lianyungang, 222005 China

**Keywords:** Dextranase, Thermostability, *Arthrobacter oxydans*, Site-directed mutagenesis, GH49

## Abstract

As an indispensable enzyme for the hydrolysis of dextran, dextranase has been widely used in the fields of food and medicine. It should be noted that the weak thermostability of dextranase has become a restricted factor for industrial applications. This study aims to improve the thermostability of dextranase AoDex in glycoside hydrolase (GH) family 49 that derived from *Arthrobacter oxydans* KQ11. Some mutants were predicted and constructed based on B-factor analysis, PoPMuSiC and HotMuSiC algorithms, and four mutants exhibited higher heat resistance. Compared with the wild-type, mutant S357P showed the best improved thermostability with a 5.4-fold increase of half-life at 60 °C, and a 2.1-fold increase of half-life at 65 °C. Furthermore, S357V displayed the most obvious increase in enzymatic activity and thermostability simultaneously. Structural modeling analysis indicated that the improved thermostability of mutants might be attributed to the introduction of proline and hydrophobic effects, which generated the rigid optimization of the structural conformation. These results illustrated that it was effective to improve the thermostability of dextranase AoDex by rational design and site-directed mutagenesis. The thermostable mutant of dextranase AoDex has potential application value, and it can also provide references for engineering other thermostable dextranases of the GH49 family.

**Supplementary Information:**

The online version contains supplementary material available at 10.1186/s13568-023-01513-2.

## Introduction

Dextran is a kind of high molecular α-glucan (Bhavani and Nisha 2010). Its main chain is made up of D-glucose molecules linked by α-(1 → 6) glycosidic bonds, and there are also possible α-(1 → 2), α-(1 → 3), or α-(1 → 4) bonds in a few branches (Díaz-Montes 2021). Dextran has broad application prospects in the food and pharmaceutical industry, and the application areas of diverse dextrans are determined by their corresponding molecular weights (Naessens et al. 2005; Xue et al. 2022). For example, medium molecular weight dextrans could be served as additives in the food industry, and low molecular weight dextrans could be used as plasma substitutes in the medical field (Falconer et al. 2011; Kothari and Goyal 2016). However, the low or medium molecular weight dextrans are obtained by hydrolysis of dextranase, which can specifically hydrolyze α-(1 → 6) glycosidic bonds of high molecular weight dextran (Khalikova et al. 2005). It was found that different dextranases belonged to multiple glycosidic hydrolase (GH) families by searching the classification in the Carbohydrate-Active Enzymes (CAZy) database (http://www.cazy.org/). According to the searching results of Protein Data Bank (https://www.rcsb.org/), only several molecular structures of dextranases were identified, and they were distributed in the GH15, GH27, GH49 and GH66 families (Larsson et al. 2003; Mizuno et al. 2004; Okazawa et al. 2015; Suzuki et al. 2016).

In addition to preparing small molecular weight dextran, dextranase has two main application directions. On the one hand, dextranase can hydrolyze dextran in a sucrose solution in quantity so as to reduce the consumption of glucose and the energy losses of the heat transfer process in the sugar industry (Purushe et al. 2012). On the other hand, dextranase is widely used to treat dental plaque. Some bacteria, such as *Streptococcus mutans*, can ferment sucrose and produce dextran in the mouth, where adherent bacteria reproduce and generate inflammation (Lai et al. 2019; Otsuka et al. 2015). Therefore, dextranase was added to toothpaste, mouthwash, and other oral hygiene products for the further prevention and treatment of dental caries (Jiao et al. 2014; Juntarachot et al. 2020). However, the industrial applications mentioned above depend mostly on the thermostability of dextranase. Both operating temperatures in sugar manufacturing processes and the long-term storage of toothpaste at ambient temperatures put higher requirements on the heat resistance of dextranase.

Some microorganisms have been reported to synthesize dextranase, such as *Thermotoga lettingae*, *Flavobacterium johnsoniae*, *Chaetomium globosum* and so forth (Gozu et al. 2016; Kim and Kim 2010; Yang et al. 2018). However, the optimal temperature for most dextranases was known to be 30–60 °C, and they could remain active for several hours. The optimal temperature of several dextranases was also reported to reach 65–70 °C, but they were originated from *Thermoanaerobacter* species, whose bacterial culture and enzyme purification were relatively complicated (Hoster et al. 2001; Park et al. 2012; Wynter et al. 1996). The dextranases available in commerce have limited thermostability and half-life. Consequently, improving the thermostability of dextranase derived from microorganisms has been a crucial project in recent years.

Previously, a dextranase AoDex secreted by *Arthrobacter oxydans* KQ11 was expressed and purified in our laboratory. It belongs to the GH49 family and has a molecular weight of 66 kDa, and exhibits excellent activity at 50–55 °C and pH 7.0–9.0 (Liu et al. 2019; Wang et al. 2014a). Furthermore, the thermostability of AoDex was tried to improve by using diversified methods. An atmospheric and room-temperature plasma (ARTP) method was used to mutate the wild-type strain, and the optimum temperature of a mutant strain was 5 °C higher than the wide strain (Wang et al. 2014b). The crystal structure of AoDex was also determined and the mutagenesis was preliminarily performed on AoDex, and the mutant S357F showed increased thermostability compared with the wild-type (Ren et al. 2019). In this study, the thermostability of AoDex was further enhanced by the site-directed mutagenesis strategy, which was based on a series of predicted software. Our results indicated that the AoDex mutants with improved thermostability could be contributed to industrial applications.

## Materials and methods

### Plasmids construction of mutant dextranases

The plasmid pCold III-KQ that contains complete gene for dextranase from *A. oxidans* KQ11 (GenBank Accession No. MK118723.1) was constructed in our previous study (Ren et al. 2019). Based on the prediction of an online tool SignalP 5.0 (Almagro Armenteros et al. 2019), the N-terminal of the full-length dextranase AoDex has a signal peptide of 32 amino acid residues. In order to realize the intracellular expression of AoDex, the signal sequence was cleaved. Subsequently, the gene segments that remove the N-terminal signal peptide sequences of dextranase were cloned and ligated into the pCold III vector, and then the new plasmid pCold III-KQ-WT was formed. Recombinant plasmids containing genes of dextranase mutants were constructed by the QuikChange method using appropriate primers and templates (Additional file 1: Table S1).

### Protein expression and purification

All recombinant plasmids that contain wild-type and mutant dextranase genes were transformed into *Escherichia coli* BL21(DE3). The strains were incubated in a constant temperature shaker at 37 °C, and when the absorbance of the bacterial cells reached approximately 0.6, isopropyl β-D-thiogalactopyranoside was added with a final concentration of 0.2 mM. The cells were then cultured on an incubator shaker at 16 °C for 24 h to induce the expression of dextranase gene.

Mutant dextranases were primarily purified by Ni^2+^ affinity chromatography. Cell pellets were centrifuged at 6000 g for 10 min and resuspended in a binding buffer of 20 mM Na_2_HPO_4_-NaH_2_PO_4_, 500 mM NaCl, 20 mM imidazole at pH 7.0. The cells were then broken by ultrasonication, and the supernatant was loaded onto a Histrap HP column (GE Healthcare, USA) after centrifugating at 13,700 g for 30 min. The target proteins were eluted with buffer containing 20 mM Na_2_HPO_4_-NaH_2_PO_4_, 500 mM NaCl, 500 mM imidazole at pH 7.0. Subsequently, the impure proteins with smaller molecular weights were removed using a centrifugal filter device (Millipore Ultra-15 30 K device, Germany). The buffer for the final dextranases was replaced with 20 mM Na_2_HPO_4_-NaH_2_PO_4_, 50 mM NaCl, 20% (v/v) glycerol at pH 7.0 for further stable preservation. All purified proteins were detected by SDS-PAGE. Protein concentration was measured using the Bradford protein assay kit (Beyotime Biotechnology, China).

### Activity assay of mutant dextranases

Dextranase activity was evaluated by the ratio of the increased concentration of reducing sugar based on the reaction between 3,5-dinitrosalicylic and the reducing sugar using dextran-20 (Sangon Biotech, China) as a substrate (Miller 1959). One unit of dextranase activity was defined as the amount of enzyme that released 1 µmol of glucose from dextran per min. Each experiment was carried out in three replicates.

### Analysis of kinetic parameters

The assays were carried out at 55 °C and pH 7.0 with three repeat experiments. The concentration of dextran was ranged from 0.05 to 1.50 mM. Kinetic parameters including *K*_m_, *v*_max_, *k*_cat_ and *k*_cat_/*K*_m_ were calculated by fitting the data to the Michaelis–Menten equation using the Linewaver-Burk plot.

### Determination of the thermostability

The thermostability of dextranase was evaluated by half-life (*T*_1/2_). Wild-type and mutant dextranases were incubated at 60 °C or 65 °C for 10–40 min at pH 7.0, and then placed on ice for 10 min. The remaining enzyme activity was measured in each case. For data processing, the untreated dextranase activity was defined as 100%, and the remaining activity was calculated as its relative enzyme activity. The inactivation kinetics of the enzymes followed the first-order reaction rate and *T*_1/2_ was calculated based on the first-order rate constant (*k*) (Ó’Fágáin 2003). Each experiment was carried out in three replicates.

### Molecular dynamics simulation

The crystal structure of AoDex was obtained from PDB 6NZS, and the designed mutants of AoDex were constructed by SWISS-MODEL (https://swissmodel.expasy.org/). The molecular dynamics (MD) simulation was performed using Gromacs version 2020.3 program with the Amber ff99sb force field. The wild-type and mutants of AoDex were executed at 328 K for 20 ns. All structures were filled with water, and counterions were added to balance the charge. The structures were then optimized through the steepest descent methods. After the simulation was complete, the root mean square deviation (RMSD) values were calculated for further analysis.

## Results

### Identification of mutation sites

The crystal structure of the dextranase AoDex had been determined (PDB: 6NZS), and several mutants with improved thermostability were obtained in our previous work (Ren et al. 2019). In this study, the systematic rational design of AoDex was further performed based on B-FITTER software, PoPMuSiC and HotMuSiC Web servers. The B-FITTER is used to analyze the B-factor value for each residue, which could reflect the flexible regions from the X-ray structure of a protein (Sun et al. 2019). The PoPMuSiC algorithm evaluates thermodynamic stability with changes in the folding free energy (ΔΔ*G*) between the wild-type and mutants (Dehouck et al. 2011). The HotMuSiC algorithm predicts the thermostability of mutated proteins by calculating the melting temperature (Δ*T*_*m*_) of each residue (Pucci et al. 2020). Consequently, the higher B-values, the more negative values of ΔΔ*G* and the more positive values of Δ*T*_*m*_ indicated more stable mutants.

**Fig. 1:**
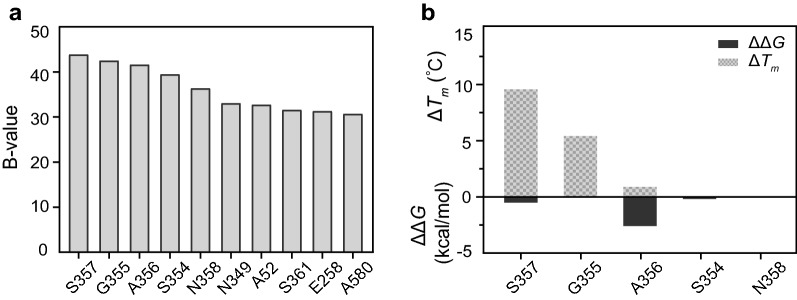
Data statistics of thermostability of dextranase AoDex with predicted software. **a** The top 10 highest B-factor values of residues calculated by the B-FITTER software. **b** Aggregate data of ΔΔ*G* (kcal/mol) and Δ*T*_*m*_ (°C) that calculated by the PoPMuSiC and HotMuSiC Web servers, respectively. The undetected values are not shown in the figure

Based on the results of the B-FITTER software, the top five B-values came from residues S354 to N358. These selected residues formed the random coil, which indicated that this region might be beneficial to improve the thermostability (Fig. 1a). Further estimation for this region from the PoPMuSiC and HotMuSiC algorithm calculated three sites (S354, A356 and S357) for mutation with both ΔΔ*G* < 0 and Δ*T*_*m*_ > 0 (Fig. 1b, Additional file 1: Table S2). Synthesizing the above results, S357 and its mutants were predicted to have the biggest B-value and Δ*T*_*m*_, respectively, and thus we identified site-saturation mutagenesis at the site of residue S357. In addition, S354 and A356 were also selected to perform some site-directed mutagenesis as putative sites to improve thermostability (Additional file 1: Table S2).

### Activity screen of mutant dextranases

**Fig. 2:**
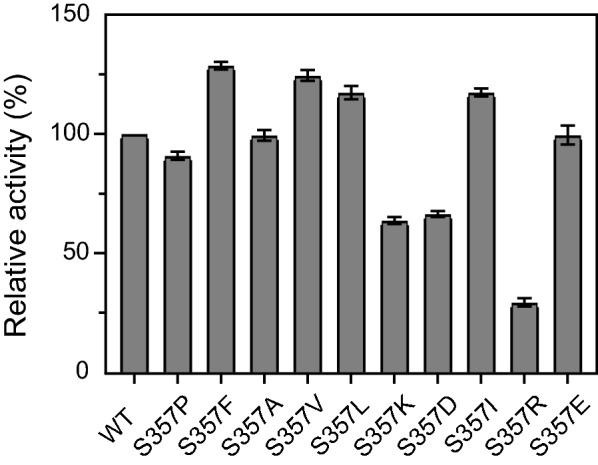
Preliminary screening of the activities of dextranase AoDex and its mutants

On the basis of the residues selected above, the mutant dextranases were successfully expressed and purified. The mutants were predicted with a molecular weight of 69 kDa, which was consistent with the results of SDS-PAGE (Additional file 1: Fig. S1). The active mutants were then preliminarily screened at 55 °C. As shown in Fig. 2, ten mutants of S357 showed enzymatic activities. The nine remaining mutants at this site tended to be inactive or precipitated (data not shown). Among the activated mutants, S357K, S357D and S357R retained less than 70% activities compared to the wild-type, hence the three mutants were not continued to be studied. Although mutants of S354Q, S354H, A356C and A356V could also be expressed, their activities were not detected at any temperature.

**Fig. 3:**
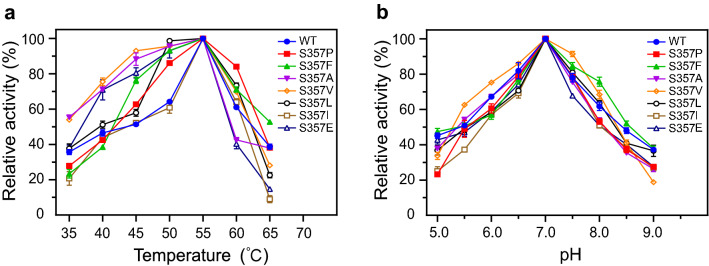
Enzymatic properties of dextranase AoDex and its mutants. Relative activity is defined as the percentage of maximum enzymatic activity under the corresponding experimental conditions. **a** The optimal temperature of AoDex and mutants. The activities were determined at pH 7.0. **b** The optimal pH of AoDex and mutants. The activities were determined at 55 °C

The optimal temperature of the active mutants was further determined within the temperature range of 35 °C to 65 °C at pH 7.0. Results showed that the optimal temperature for these mutants was still 55 °C, which was the same as the wild-type (Fig. 3a). However, some mutants (S357V, S357L, S357P, and S357F) exhibited higher activity than wild-type in the temperature range of 55–60 °C. Compared to other mutants, S357P retained more than 80% activity at 60 °C, indicating that the thermostability of S357P appeared to be higher than other mutants. In addition, the optimal pH of the active mutants was also measured within the pH range from 5.0 to 9.0 at the optimal temperature. Changes in the amino acid residue at S357 also did not affect the optimal pH of AoDex, and individual mutants such as S357V showed greater activity in the pH range of 6.0–8.0 (Fig. 3b).

### Effect of mutated residues on thermostability

**Fig. 4:**
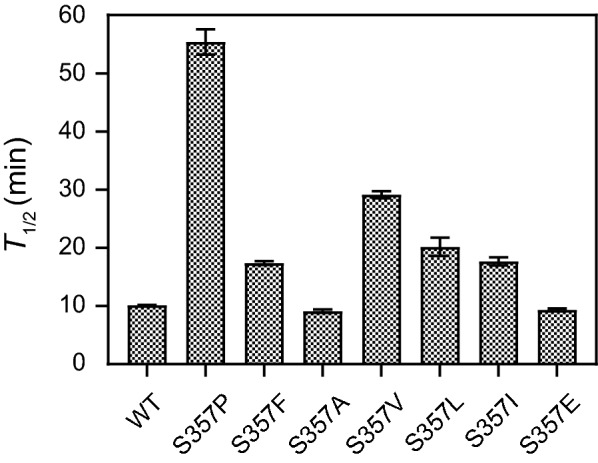
Parameters of thermostability of AoDex and the active mutants. The *T*_1/2_ values were detected at 60 °C and pH 7.0

**Fig. 5:**
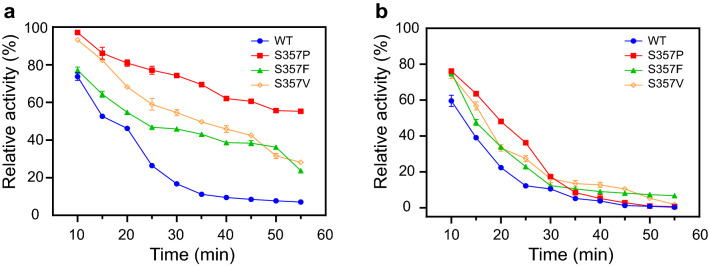
Thermostability of the wild-type and mutant dextranase AoDex during the incubation at 60 °C **a** and 65 °C **b**. Relative activity is defined as the percentage of maximum enzymatic activity under the corresponding experimental conditions. The activities were determined at pH 7.0

The thermostability of the active mutant dextranases was measured at 60 °C (Fig. 4). Results showed that the half-lives (*T*_1/2_) of S357P, S357V, S357I and S357L were longer than those of the wild-type, indicating the enhanced thermostability of these mutants. S357A and S357E displayed similar or lower *T*_1/2_ values compared to the wild-type, illustrating that the thermostability did not improve although they were active. Among the four mutants with increased thermostability, S357P and S357V showed higher *T*_1/2_ values, which were about 5.4 and 2.9 times of the wild-type, respectively. Previous studies had shown that the mutant S357F was also more stable than the wild-type, so the thermostability of S357F was reassessed. As shown in Fig. 4 and Table 1, the *T*_1/2_ value of S357F was higher than the wild-type, which was consistent with our previous results. However, S357F had a lower *T*_1/2_ value than S357P and S357V, indicating that S357P and S357V had significantly improved in the aspect of heat resistance. Furthermore, we also measured the half-lives of S357P, S357F and S357V at 65 °C, and the three mutants showed higher *T*_1/2_ values than the wild-type (Table 1). S357P exhibited the maximum value of *T*_1/2_ of 14.0 min, which was 2.1 times of the wild-type. Meanwhile, the time course of the activities for these mutants during the incubation at 60 °C and 65 °C were shown in Fig. 5. After incubating at 60 °C for 55 min, S357P performed best heat-resistance, and retained more than 55% of the initial enzymatic activity compared with other mutants (Fig. 5a). S357V showed higher residual activity than S357F in 45 min (Fig. 5a). When the incubation temperature reached 65 °C, the activities of all the enzymes exhibited dramatic declines, but the residual activity of S357P was still higher than other mutants within 25 min (Fig. 5b). To further assess the thermostability of S357P and S357V, MD simulations were performed at 328 K for 20 ns. Results showed that the structures including wild-type, S357P and S357V tended to reach the stable states with the RMSDs of 0.05–0.15 nm (Additional file 1: Fig. S2). After 5 ns simulation, the overall structural fluctuation of the mutants was less than that of the wild-type, suggesting that these mutants had better thermostability than wild-type. The mutant S357V showed an average RMSD value of 0.110, while S357P displayed a lower RMSD value of 0.106, indicating that S357P was more stable than S357V, and the results basically matched their half-life experiments. Therefore, S357P exerted the property with the highest thermostability of these mutants despite the slightly lower activity.

**Table 1:** Specific activities and half-lives of wild-type and mutant dextranase AoDex

Dextranase	Specific activity (U/mg)	*T*_1/2_ at 60 °C (min)	*T*_1/2_ at 65 °C (min)
Wild-type	859 ± 9.2	10.2 ± 0.1	6.8 ± 0.1
S357P	780 ± 6.1	55.4 ± 2.2	14.0 ± 0.2
S357F	1104 ± 6.1	17.4 ± 0.3	9.0 ± 0.1
S357V	1069 ± 10.8	29.6 ± 0.1	9.9 ± 0.4

### Enzymatic characterizations of the thermostable mutants

The kinetic parameters of the mutants with improved thermostability were determined at the optimal temperature of 55 °C. As shown in Table 2, the *K*_m_ values of the mutants increased to different degrees, suggesting that all of these mutant dextranases reduced affinity for the substrate of dextran-20. The *k*_cat_ values of S357V and S357I improved 1.6 and 1.2 times, respectively, and showed enhanced catalytic rate constants. However, the *k*_cat_ values of S357P and S357L were similar or slightly decreased to the wild-type. S357V exhibited a maximal *k*_cat_/*K*_m_ value, which means that it had a higher catalytic efficiency. We also compared the kinetics of S357F with other mutants, and it was found that although the catalytic efficiency of S357F was increased, the affinity for the substrate decreased significantly. From the above results, it was concluded that the thermostable mutant of S357P showed a decreased affinity for substrates and a lower catalytic efficiency, but remained higher thermostability. Although the thermostability of S357V was lower than that of S357P, its catalytic activity increased significantly. Therefore, mutant S357V could enhance the thermostability and catalytic efficiency of dextranase synchronously.

**Table 2:** The kinetic parameters of wild-type and mutant dextranase AoDex

Dextranase	*v*_max_ (mmol·L^−1^·min^−1^)	*K*_m_ (μmol·L^−1^)	*k*_cat_ (s^−1^)	*k*_cat_/*K*_m_ (μmol^−1^·L·s^−1^) × 10^3^
Wild-type	2.83 ± 0.05	50.9 ± 1.5	12.6 ± 0.2	247.8 ± 8.3
S357P	4.83 ± 0.04	62.0 ± 1.3	11.1 ± 0.1	179.1 ± 4.1
S357F	4.71 ± 0.15	115.6 ± 6.6	25.0 ± 0.8	217.0 ± 14.2
S357V	4.26 ± 0.05	58.0 ± 1.5	20.3 ± 0.2	350.2 ± 9.7
S357I	5.84 ± 0.02	121.5 ± 1.4	14.7 ± 0.1	121.0 ± 1.6
S357L	4.89 ± 0.11	128.4 ± 5.2	12.8 ± 0.3	99.9 ± 4.6

## Discussion

Improving the thermostability of enzymes has become a hot and difficult issue of enzymology. Enzymes with high heat resistance could be more conducive to their stable preservation and promote their application in related fields. Generally, the factors affecting the thermostability of proteins mainly include the non-covalent interactions of residues such as ionic bonds, hydrogen bonds and hydrophobic interactions, and some covalent binding such as disulfide bonds (Xu et al. 2020). Additionally, rigid regions in proteins may be crucial for maintaining thermostability (Radestock and Gohlke 2011). Therefore, in this study, reasonable predictions of flexible sites for dextranase AoDex were made using relevant software and Web servers to investigate strategies to improve the thermostability of dextranase.

**Fig. 6:**
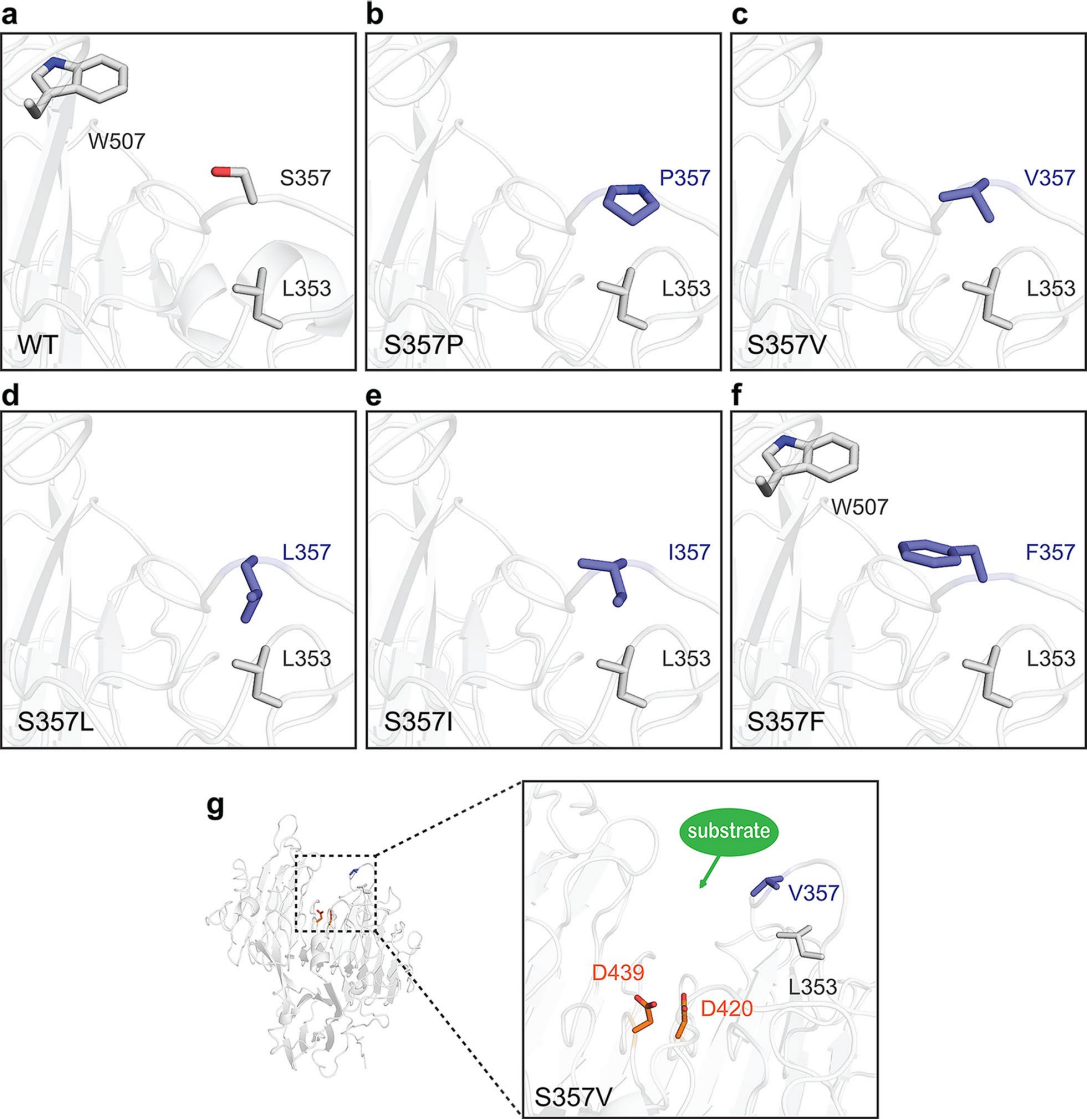
Changes in intramolecular interactions of dextranase AoDex that are caused by mutants at residue S357. The structural models of the mutants were determined by SWISS-MODEL. The ribbon representation of dextranase is shown in gray. The mutant residues are labeled as navy-blue sticks. The predicted catalytic residues are labeled as orange sticks. **a** The relative positions of key residues of the wild-type. **b**–**f** Relative positions of key residues of AoDex mutants. **g** The overview of relative positions of substrate binding channels, catalytic residues, and mutant residues (take mutant V357 for example)

The above results suggested that S357P was the most stable dextranase among heat-resistance mutants, indicating that the introduction of proline significantly improved thermostability. Proline contains a pyrrolidine ring on its side chain, resulting in its special rigid conformation (Allen et al. 2004). Based on the structural and statistical analysis, the thermostability of a protein could be improved through rigidifying the flexible regions by introducing prolines to the structure (Arnold and Raines 2016; Xie et al. 2020; Yu et al. 2015). Besides, the positions where residues were replaced could also affect the thermostability of the protein. Studies showed that it was more conducive to improve the thermostability when proline replaced other amino acids in the second positions of β-turns or N1 positions of α-helices (Trevino et al. 2007; Xu et al. 2020). Furthermore, prolines in loop regions played a significant role in maintaining the thermostability (Farhat-Khemakhem et al. 2013; Yu et al. 2015). In this study, the substituted position of proline for S357 in AoDex is located in an exposed long loop between two β-sheets of the catalytic domain, as well as at the entrance of the substrate binding channel. This unique location of proline may lead to a sharp bend in the peptide chain; hence, it may help rigidify flexible regions of the dextranase AoDex, or form the hydrophobic interaction between its own side chain and other hydrophobic residues, thus increasing the rigidity of the peptide chains and making the structure more compact (Fig. 6b). The replacement of proline in flexible regions provided new possibilities for the thermostable modification of dextranases. The mutant S357F could enhance thermostability, which was also verified in our previous study. The substitution of phenylalanine was analyzed to form an aromatic interaction with surrounding aromatic amino acids such as W507 (Fig. 6f) (Ren et al. 2019). As is known, the side chains of valine, leucine, isoleucine, and phenylalanine are all hydrophobic. In this study, the thermostability of S357V, S357I and S357L was also improved, although these mutants were less heat resistant than S357P and S357F. According to the structural model of single point mutation for S357, the hydrophobic residues replaced serine and formed new hydrophobic interactions with adjacent L353, which would probably increase the thermostability of dextranase (Fig. 6c, d, e). Thus, the improved heat resistance of mutant S357F could also be attributed to increase hydrophobic forces. Furthermore, the thermostability of S357A and S357E was similar to that of the wild-type, suggesting that the interactions generated by the replacement of the two residues have little effect on the overall structural rigidity.

In addition, an increase in the activity of mutant dextranases was also found in this study. Based on our previous studies, the catalytic residues of the dextranase AoDex were predicted to be D420 and D439, and the substrate channel tended to form in the void outside the catalytic domain (Ren et al. 2019). The structure of AoDex showed that the mutant site of S357 was just close to the entrance of the substrate channel. When the mutants of S357 generated hydrophobic interactions with adjacent L353, the size and shape of the substrate channel could be changed. And it probably promoted the binding of substrates and catalytic residues, which also might explain the reason for the increased activity of mutants S357V, S357I, S357L and S357F (Fig. 6g). S357F was slightly more active than S357V, indicating that the conformation of phenylalanine might be more favorable for substrate binding than other hydrophobic residues. Valine had a shorter side chain compared to leucine and isoleucine, and, in the meantime, S357V had higher enzymatic activity compared to S357I and S357L. Therefore, it appeared that the length of the side chain of an amino acid could also change the shape of the substrate binding channel and then affect the catalytic activity. In addition, the activities of S357A and S357E were similar to the wild-type, demonstrating that these substitutions of residues did not appear to affect the flexibility of the loop. However, the activity of S357P with excellent thermostability decreased slightly. It was previously reported that the increase in the thermostability of enzymes was usually accompanied by the decrease in the activities, which might explain the reason for the above results (Xie et al. 2014). S357K, S357D and S357R with decreased activities illustrated that the structural conformation of the three mutations significantly affected catalytic activities.

Currently, there are several strategies to obtain the heat resistant dextranases. One way is to screen the thermophilic dextranases of thermophiles (Hoster et al. 2001; Park et al. 2012). Nonetheless, both the rigorous culture conditions of thermophilic microorganisms and the limited stability of natural enzymes put higher requirements on the studies. Another option is to achieve the thermostability of dextranases by directed evolution or rational design, and these techniques have been widely used in other multiple enzymes such as xylanases and proteinases (Rigoldi et al. 2018). Several variants of a dextranase that originated from *Paenibacillus* sp. had been reported to increase the half-lives by 2.3–6.9 times through random mutagenesis (Hild et al. 2007). A GH97 dextranase from *Pseudoalteromonas* sp. K8 increased thermostability at 33 °C by rational design (Zhang et al. 2020). Presently, there have been few studies on the thermostability of dextranase, and this is probably due to the fact that there are not many dextranases whose structures have been resolved. Except for dextranase AoDex, the other GH49 dextranase with a known structure was Dex49A. It was derived from *Penicillium minioluteum*, and its three-dimensional structure resembled that of AoDex (Larsson et al. 2003; Ren et al. 2019). Compared with Dex49A, AoDex was found to have several extended loops on the surface of the structure. Moreover, the residues of AoDex from S354 to N358 that were predicted to be beneficial to improve thermostability were also located in these exposed loop regions, and this feature was absent in the structure of Dex49A. It has been reported that some deletions in the exposed loop regions of a thermophilic protein are more likely to help to lower its unfolding entropy and increase the thermostability (Suzuki et al. 2016; Thompson and Eisenberg 1999). The mutations of S357 from the loop regions might be speculated to result in the broken of the conformational entropy of the original structure, and thus the thermostability of relevant mutants was improved. Based on the structure of Dex49A, a GH49 dextranase that originated from *Lipomyces starkeyi* was modeled, and enhanced its optimal temperature by introducing disulfide bonds (Chen et al. 2009). For the dextranase of *P.minioluteum*, studies had found that the recombinant expression of dextranase in *Pichia pastoris* could also significantly improve thermostability (Beldarrain et al. 2003). The above results were specific to the dextranase Dex49A, which was derived from fungi. Dextranase AoDex also belonged to GH49, and a favorable mutant S357F had been mined in our previous research. In this study, we further attempted to improve the thermostability of dextranase AoDex by rational design and obtained several mutants with better heat resistance, including S357P and S357V. Enzymes of the same glucoside hydrolase have the similar substrate binding pocket and catalytic mechanism; hence, the findings of AoDex could also provide some references for the thermostability of other dextranase in GH49.

## Supplementary Information

**Additional file 1. Table S1** Primer sequences used for plasmids mutagenesis in this study. **Table S2** Data statistics of selected mutants of dextranase AoDex from B-FITTER, PoPMuSiC and HotMuSiC. **Figure. S1** SDS-PAGE of the wild-type (WT) and mutants of dextranase AoDex. **Figure. S2** The RMSD values of the wild-type and mutants of AoDex at 328 K.

## Data Availability

The datasets generated during and/or analyzed during the current study are available from the corresponding author on reasonable request.

## Abbreviations

CAZy: Carbohydrate-active enzymes
GH: Glycoside hydrolase
WT: Wild type
SDS-PAGE: Sodium dodecyl-sulfate polyacrylamide gel electrophoresis
RMSD: Root mean square error
