# Supplementary material for: Improving the thermostability of GH49 dextranase AoDex by site-directed mutagenesis
Source: AMB Express. 2023 Jan 19;13:7. doi: 10.1186/s13568-023-01513-2 (PMC9852402; doi:10.1186/s13568-023-01513-2)
Supplement: Supplementary file 1 — Additional file 1. Table S1 Primer sequences used for plasmids mutagenesis in this study. Table S2 Data statistics of selected mutants of dextranase AoDex from B-FITTER, PoPMuSiC and HotMuSiC. Figure. S1 SDS-PAGE of the wild-type (WT) and mutants of dextranase AoDex. Figure. S2 The RMSD values of the wild-type and mutants of AoDex at 328 K. [file 13568_2023_1513_MOESM1_ESM.pdf]

AMB Express

Additional file 1 for

Improving the thermostability of GH49 dextranase AoDex by site-directed mutagenesis

Zhen Wei[1,3*], Jinling Chen[2], Linxiang Xu[1,3], Nannan Liu,[1,3] Jie Yang[1,2], Shujun Wang[1,2*]

[1]Jiangsu Key Laboratory of Marine Bioresources and Environment, Co-Innovation Center of Jiangsu Marine Bio-industry Technology, Jiangsu Ocean University, Lianyungang 222005, China.

[2]School of Food Science and Engineering, Jiangsu Ocean University, Lianyungang 222005, China.

[3]Jiangsu Institute of Marine Resources Development, Jiangsu Ocean University, Lianyungang 222005, China.

[*] Corresponding authors. E-mail address: 2019000036@jou.edu.cn (Zhen Wei), shjwang@hhit.edu.cn (Shujun Wang)

Additional file Table Legends

Table S1 Primer sequences used for plasmids mutagenesis in this study.

Table S2 Data statistics of selected mutants of dextranase AoDex from B-FITTER, PoPMuSiC and HotMuSiC.

Table S1 Primer sequences used for plasmids mutagenesis in this study.

Type of mutants	Primer sequences[a]	Plasmid templates
S354Q	F,5'-CAATAACAACTACAACCACCTCCAAGGAGCGTCCAACTGCCAC-3'<br>R,5'-GTGGCAGTTGGACGCTCCTTGGAGGTGGTTGTAGTTGTTATTG-3'	pCold III-KQ-WT
S354H	F,5'-CAATAACAACTACAACCACCTCCACGGAGCGTCCAACTGCCAC-3'<br>R,5'-GTGGCAGTTGGACGCTCCGTGGAGGTGGTTGTAGTTGTTATTG-3'	pCold III-KQ-WT
A356C	F,5'-CAACCACCTCAGCGGATGCTCCAACTGCCACTCGTCGTG-3'<br>R,5'-CACGACGAGTGGCAGTTGGAGCATCCGCTGAGGTGGTTG-3'	pCold III-KQ-WT
A356V	F,5'-CAACCACCTCAGCGGAGTGTCCAACTGCCACTCGTCGTG-3'<br>R,5’-CACGACGAGTGGCAGTTGGACACTCCGCTGAGGTGGTTG-3'	pCold III-KQ-WT
S357P	F,5'-CAACCACCTCAGCGGAGCGCCCAACTGCCACTCGTCGTGCG-3'<br>R,5'-CGCACGACGAGTGGCAGTTGGGCGCTCCGCTGAGGTGGTTG-3'	pCold III-KQ-WT
S357D	F,5'-CAACCACCTCAGCGGAGCGGACAACTGCCACTCGTCGTGCGTG-3'<br>R,5'-CACGCACGACGAGTGGCAGTTGTCCGCTCCGCTGAGGTGGTTG-3'	pCold III-KQ-WT
S357A	F,5'-CCACCTCAGCGGAGCGGCCAACTGCCACTCGTCGTGCGTG-3'<br>R,5'-CACGCACGACGAGTGGCAGTTGGCCGCTCCGCTGAGGTGG-3'	pCold III-KQ-WT
S357N	F,5'-CAACCACCTCAGCGGAGCGAACAACTGCCACTCGTCGTGCGTG-3'<br>R,5'-CACGCACGACGAGTGGCAGTTGTTCGCTCCGCTGAGGTGGTTG-3'	pCold III-KQ-WT
S357V	F,5'-CAACCACCTCAGCGGAGCGGTCAACTGCCACTCGTCGTGCGTG-3'<br>R,5'-CACGCACGACGAGTGGCAGTTGACCGCTCCGCTGAGGTGGTTG-3'	pCold III-KQ-WT

Type of mutants	Primer sequences[a]	Plasmid templates
S357T	F,5'-CAACCACCTCAGCGGAGCGACCAACTGCCACTCGTCGTGCGTG-3'<br>R,5'-CACGCACGACGAGTGGCAGTTGGTCGCTCCGCTGAGGTGGTTG-3'	pCold III-KQ-WT
S357M	F,5'-CAACCACCTCAGCGGAGCGATGAACTGCCACTCGTCGTGCGTG-3'<br>R,5'-CACGCACGACGAGTGGCAGTTCATCGCTCCGCTGAGGTGGTTG-3'	pCold III-KQ-WT
S357L	F,5'-CAACCACCTCAGCGGAGCGCTCAACTGCCACTCGTCGTGCGTG-3'<br>R,5'-CACGCACGACGAGTGGCAGTTGAGCGCTCCGCTGAGGTGGTTG-3'	pCold III-KQ-WT
S357H	F,5'-CAACCACCTCAGCGGAGCGCACAACTGCCACTCGTCGTGCG-3'<br>R,5'-CGCACGACGAGTGGCAGTTGTGCGCTCCGCTGAGGTGGTTG-3'	pCold III-KQ-WT
S357Q	F,5'-CAACCACCTCAGCGGAGCGCAAAACTGCCACTCGTCGTGCGTG-3'<br>R,5'-CACGCACGACGAGTGGCAGTTTTGCGCTCCGCTGAGGTGGTTG-3'	pCold III-KQ-S357H
S357I	F,5'-CAACCACCTCAGCGGAGCGATCAACTGCCACTCGTCGTGCGTG-3'<br>R,5'-CACGCACGACGAGTGGCAGTTGATCGCTCCGCTGAGGTGGTTG-3'	pCold III-KQ-WT
S357E	F,5'-CAACCACCTCAGCGGAGCGGAGAACTGCCACTCGTCGTGCGTG-3'<br>R,5'-CACGCACGACGAGTGGCAGTTCTCCGCTCCGCTGAGGTGGTTG-3'	pCold III-KQ-S357D
S357R	F,5'-CAACCACCTCAGCGGAGCGCGCAACTGCCACTCGTCGTGCGTG-3'<br>R,5'-CACGCACGACGAGTGGCAGTTGCGCGCTCCGCTGAGGTGGTTG-3'	pCold III-KQ-WT
S357K	F,5'-CAACCACCTCAGCGGAGCGAAGAACTGCCACTCGTCGTGCGTG-3'<br>R,5'-CACGCACGACGAGTGGCAGTTCTTCGCTCCGCTGAGGTGGTTG-3'	pCold III-KQ-S357M

Type of mutants	Primer sequences[a]	Plasmid templates
S357F	F,5'-CAACCACCTCAGCGGAGCGTTCAACTGCCACTCGTCGTGCGTG-3'<br>R,5'-CACGCACGACGAGTGGCAGTTGAACGCTCCGCTGAGGTGGTTG-3'	pCold III-KQ-WT
S357Y	F,5'-CAACCACCTCAGCGGAGCGTACAACTGCCACTCGTCGTGCGTG-3'<br>R,5'-CACGCACGACGAGTGGCAGTTGTACGCTCCGCTGAGGTGGTTG-3'	pCold III-KQ-WT
S357W	F,5'-CAACCACCTCAGCGGAGCGTGGAACTGCCACTCGTCGTGCG-3'<br>R,5'-CGCACGACGAGTGGCAGTTCCACGCTCCGCTGAGGTGGTTG-3'	pCold III-KQ-WT
S357C	F,5'-CAACCACCTCAGCGGAGCGTGCAACTGCCACTCGTCGTGCG-3'<br>R,5'-CGCACGACGAGTGGCAGTTGCACGCTCCGCTGAGGTGGTTG-3'	pCold III-KQ-WT
S357G	F,5'-CAACCACCTCAGCGGAGCGGGCAACTGCCACTCGTCGTGCGTG-3'<br>R,5'-CACGCACGACGAGTGGCAGTTGCCCGCTCCGCTGAGGTGGTTG-3'	pCold III-KQ-S357C

[a]The mutated bases are underlined.

Table S2 Data statistics of selected mutants of dextranase AoDex from B-FITTER, PoPMuSiC and HotMuSiC.

Residue	B-value[a]	Mutant	$\Delta\Delta G$ (kcal/mol) [a]	ΔT_m (°C) [a]
S354	39.45	S354Q	-0.15	N.D.[b]
		S354H	N.D.[b]	0.07
A356	41.60	A356C	-0.74	0.27
		A356V	-0.43	0.26
S357	43.83	S357P	-0.52	0.54
		S357D	-0.06	0.23
		S357A	-0.1	N.D.[b]
		S357N	N.D.[b]	0.07
		S357V	N.D.[b]	0.20
		S357T	N.D.[b]	0.21
		S357M	N.D.[b]	0.27
		S357L	N.D.[b]	0.62
		S357H	N.D.[b]	0.52
		S357Q	N.D.[b]	0.54
		S357I	N.D.[b]	0.55
		S357E	N.D.[b]	0.62
		S357R	N.D.[b]	0.76
		S357K	N.D.[b]	0.79
		S357F	N.D.[b]	1.04
		S357Y	N.D.[b]	1.33
		S357W	N.D.[b]	1.60
		S357C	N.D.[b]	N.D.[b]
		S357G	N.D.[b]	N.D.[b]

[a]The data of B-value, $\Delta\Delta G$ (kcal/mol) and ΔT_m (°C) were calculated by B-FITTER, PoPMuSiC and HotMuSiC, respectively.

[b]N.D. represented that this value was not detected by corresponding algorithm.

Additional file Figure Legends

Fig. S1 SDS-PAGE of the wild-type (WT) and mutants of dextranase AoDex.

Fig. S2 The RMSD values of the wild-type and mutants of AoDex at 328 K.

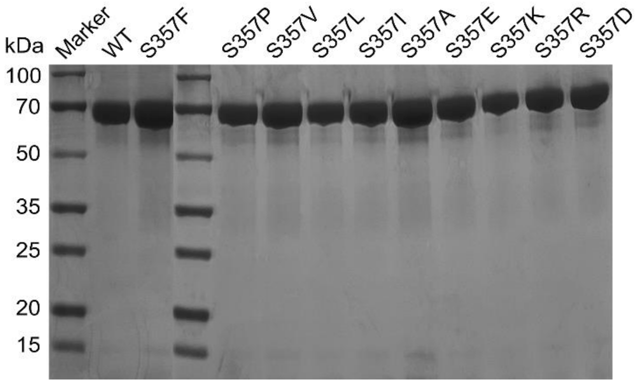

Fig. S1 SDS-PAGE of the wild-type (WT) and mutants of dextranase AoDex.

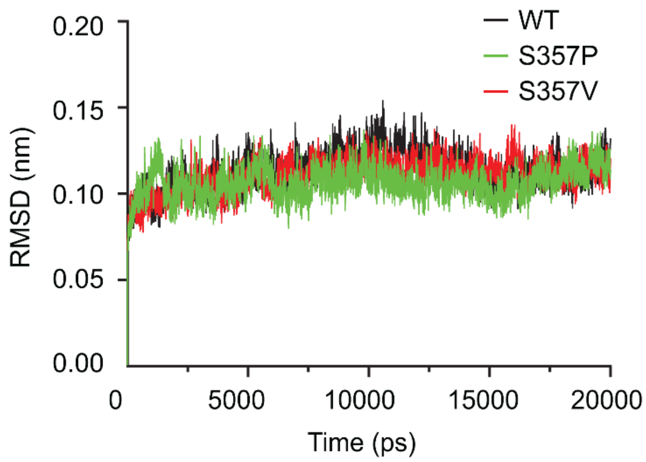

Fig. S2 The RMSD values of the wild-type and mutants of AoDex at 328 K.
